# A Diagnostic Case Study for Manufacturing Gas-Phase Chemical Sensors

**DOI:** 10.3390/chemosensors12080155

**Published:** 2024-08-07

**Authors:** Raquel Pimentel Contreras, Dylan T. Koch, Patrick Gibson, Mitchell M. McCartney, Bradley S. Chew, Pranay Chakraborty, Daniel A. Chevy, Reid Honeycutt, Joseph Haun, Thomas Griffin, Tristan L. Hicks, Cristina E. Davis

**Affiliations:** 1 Department of Mechanical and Aerospace Engineering, University of California Davis, Davis, CA, USA; 2 UC Davis Lung Center, One Shields Avenue, Davis, CA 95616, USA.; 3 VA Northern California Health Care System, 10535 Hospital Way, Mather, CA 95655, USA; 4 Department of Electrical Engineering, University of California Davis, Davis, CA, USA

**Keywords:** Natural Gas, Odorants, Gas Chromatography, Chemical Sensors, Mercaptans

## Abstract

In this work, we describe the design, manufacturing development and refinement of a chemical detection platform designed to identify specific odorants in the natural gas industry. As the demand for reliable and sensitive volatile organic compound (VOC) detection systems grows, our project aimed to construct multiple prototypes to enhance detection capabilities and provide portable detection platforms. Throughout the development process across nominally identical and duplicate instruments, various failure modes were encountered, which provided insight into the design and manufacturing challenges present when designing such platforms. We conducted a post-hoc root cause analysis for each failure mode, leading to a series of design modifications and solutions. This paper details these design and manufacturing challenges, the analytical methods used to diagnose and address them, and the resulting improvements in system performance. In the end, a debugging flow chart is presented to aid future researchers in solving possible issues that could be encountered. Our findings show the complexities of bespoke chemical sensor design for unique applications and highlight the critical importance of iterative testing and problem-solving in the development of industrial detection technologies. Achieving consistency across devices is essential for optimizing device-to-device efficiency. The work presented is the first step towards ensuring uniform performance across a production run of chemically sensitive devices. In the future a universal device calibration model will be implemented, eliminating the need to collect data from each individual device.

## 1. Introduction

Recent advancements in portable volatile organic compound (VOC) detectors have based on gas chromatography (GC) and/or differential mobility spectrometry (DMS) technologies led to the development of numerous applications, ranging from the detection of pollutants in ambient air to monitoring biomarkers in clinical settings [1–4]. Chemical sensing varied across different fields: environmental monitoring focuses on pollutant detection [5, 6], clinical applications often involve health-related biomarkers [3, 7] and industrial uses can include quality control manufacturing processes. [4, 8] For instance, (Fabianowski et. al. [1] demonstrated the detection and identification of VOCs using differential mobility spectrometry (DMS), while Fraustro-Vicencio et al. [2]characterized the performance of a compact gas chromatograph-photoionization detector (GC-PID) for near-real-time analysis and field deployment of benzene, toluene ethylbenzene, and xylene (BTEX) compounds. Gunter et al. [3] explored breath sensors for health monitoring, and Camara et al. [4] detected and quantified natural contaminants in wine using gas chromatography-differential mobility spectrometry (GC-DMS). Other chemical sensing technologies have also been used in environmental monitoring. Metal oxide sensors are often chosen for their sensitivity and accuracy. However, they typically suffer from low selectivity and are limited by operating temperature range, humidity variations, and recovery time, as noted by Tereshkov et al. [9] To address some of these limitations, Li et al. [10] developed a novel NH_3_ sensor using Pd-decorated ZnO hexagonal microdiscs, showcasing enhanced sensitivity and selectivity in ammonia detection. NonNDIR detectors are frequently used to sense trace pollutants in gas mixtures, but have historically had limitations with sensitivity and cross-interference. Zhang et al. [11] developed a high-sensitivity ethylene gas sensor that addressed the sensitivity limitations with limit of detection as low as 1 ppm as compared to 25 to 34 ppm from previous non-dispersive infrared (NDIR) sensors. Xu et al. [12] introduced a multi-gas detection system based on NDIR spectral technology, which provided an effective method for detecting multiple gases simultaneously and demonstrate good resistance to cross-interference. Previously, our group has developed a prototype portable VOC detector designed for enhanced sensitivity and selectivity based on GC-DMS technologies [13]. Building upon the prototype design, we are constructing a fleet of VOC sensitive devices capable of field deployment and a quality assessment (QA)-quality control (QC) process to accompany this work. At present, this work addresses the first steps to establish uniformity across production devices by establishing a standardized test and evaluation (T&E) methodology for chemical sensors.

Despite the growing applications of these categories of devices [5, 8] the literature remains limited on methodologies for troubleshooting and enhancing chemical sensor system reliability during manufacturing and assembly. There is little guidance on how to systematically compare the performance of multiple seemingly-identical copies of the same chemical sensor device design. Studies typically focus on the development and demonstration of a single prototype, leaving a gap in knowledge regarding the mass production and quality assurance of such devices [6, 7]. Integration of complex systems relying on many components and sub-systems for it to function can be difficult to execute. Building upon the foundation of our earlier work led by Fung, et. al. [13], this work explores the challenges of scaling from a single prototype to multiple operational units. We present a comprehensive test and evaluation (T&E) approach to the quality assessment/quality control (QA/QC) processes essential for ensuring consistent device performance. Five VOC sensor units were constructed, verifying component performance and integrated device performance along the way. Our objective is to provide a replicable process that can aid researchers and developers in enhancing the reliability and reproducibility of portable chemical detection technologies.

## 2. Materials and Methods

### 2.1. Device System Overview

**Figure 1:**
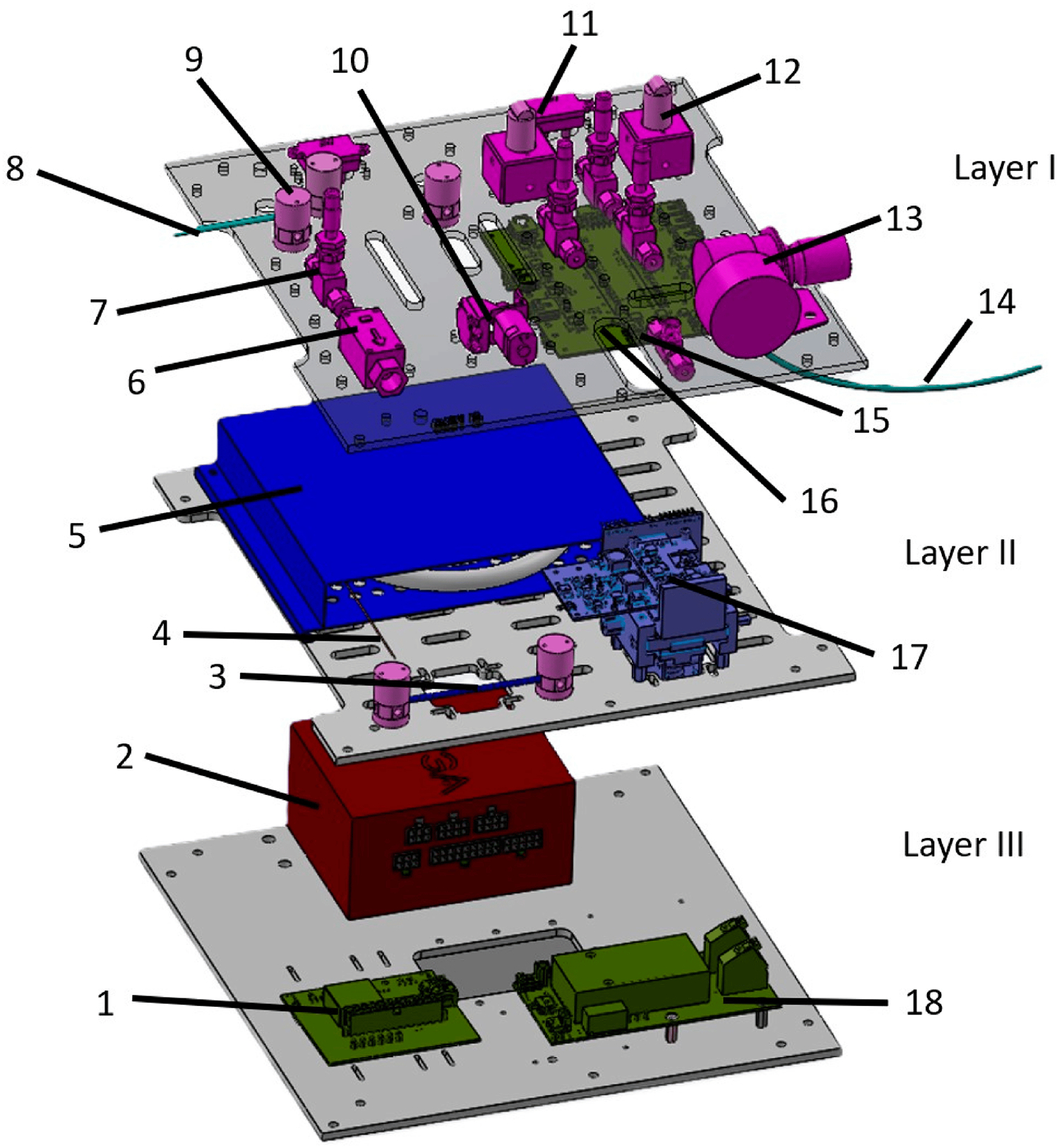
System layout of the Three main sub-systems of the chemical detection device. Layer I, layer II, and layer III are the flow control, chemical analysis, electronics/power sub-systems, respectively. Each of the parts are numbered as follows: (1) main power electronics, (2) power supply, (3) sorbent trap, (4) heated line (HL), (5) Gas chromatography columns, 6 odorant sample flow sensor, 7 needle valve, 8 odorant sample inlet, 9 3-way valve, 10 odorant sample pump, 11 nitrogen flow sensor, 12 proportional valve, 13 pressure regulator, 14 nitrogen inlet, 15 metal tee, 16 device control electronics, 17 differential mobility spectrometer, 18 ionization power electronics.

Our group previously reported a single prototype chemical detection platform for natural gas odorants [13]. Similarly, the device reported in this study can be broken down into three major sub-sections: the flow system, the detection system, and electronics. The flow system consists of pressure regulators (McMaster, 6763K81) figure 1, number13, proportional valves (Norgren, D170.0004) figure 1, number12, needle valves (Swagelok, SS-SS2) figure 1, number 7, flow sensors (Honeywell, HAFBLF0750C4AX5, HAFBLF0050CAAX5, and OMRON, D6F-01N2–000) figure 1, number 6 and 11, and 3-way valves (Clippard, NR1–3M-12) figure 1, number9 that work together to regulate flows as necessary to sweep chemicals through the various stages of chemical analysis. The detection system consists of the sorbent trap, GC Column (Agilent, 123–1334LTM) figure 1, number 5, and the differential mobility spectrometer detector (figure 1, number17). While the GC column is commercially available, the sorbent trap (figure 1, number3) and detector are custom components manufactured at UC Davis and detailed in our prior publication [13]. The electronics consists of main power electronics figure 1, number1, main control electronics with an Arduino based Teensy 3.6 microcontroller (figure 1, number16), the ionization power board (figure 1, number18), and the device power supply (figure 1, number2).

Between iterations significant modifications were made to the device improving functionality. Principally, the gas-flow controlled system for this iteration was significantly overhauled to improve performance. Long-term validation revealed the mechatronic actuators and sensors responsible for metering a precisely controlled volumetric flow rate were inadequately specified for this task. A traditional mechanical valve was installed in place of the digitally controlled flow system for greater reliability and tunability at low flow rates. In brief, the odorant sensor is a portable platform to measure odorants from natural gas samples. The device can be connected directly to a pressurized natural gas line, or the sample can be introduced from a sampling canister like Tedlar bags.

Sample analysis is performed in three stages. A sorbent trap, custom made by the UC Davis team to extract odorants from natural gas; a gas chromatography (GC) column, which separates the odorant species so they can be measured individually; and the differential mobility spectrometer (DMS) detector, which measures the odorant abundance. The device measures six chemicals commonly used as natural gas odorants. Tetrahydrothiophene (THT), Isopropyl Mercaptan (IPM), N-Propyl Mercaptan (NPM), Tert-Butyl Mercaptan (TBM), Ethanethiol (ETM), and Dimethyl Sulfide (DMS-odorant). Test processed with our laboratory AnalyzeIMS or “AIMS” Software [14–16] which converts detector data into a concentration of each mercaptan.

### 2.2. Case Presentation-Device Components:

We present an overview of the device components, and report component-level performance data prior to device sub-system assembly. In the following section, we demonstrate experimental result errors observed during assembly of 5 units, and detail our troubleshooting approach. In total, 5 units were constructed and had full component level testing. A system level layout of the chemical sensor system is show (Figure 1).

Odorants collected in the sample need to be directed through various sub-systems within the device for analysis. Transportation of analytes to these stages is controlled by the flow system, consisting of the previously detailed individual components, which generates and controls a flow of nitrogen carrier gas, working in unison to regulate flows as necessary to direct odorants through the analysis pathway [13].

**Figure 2:**
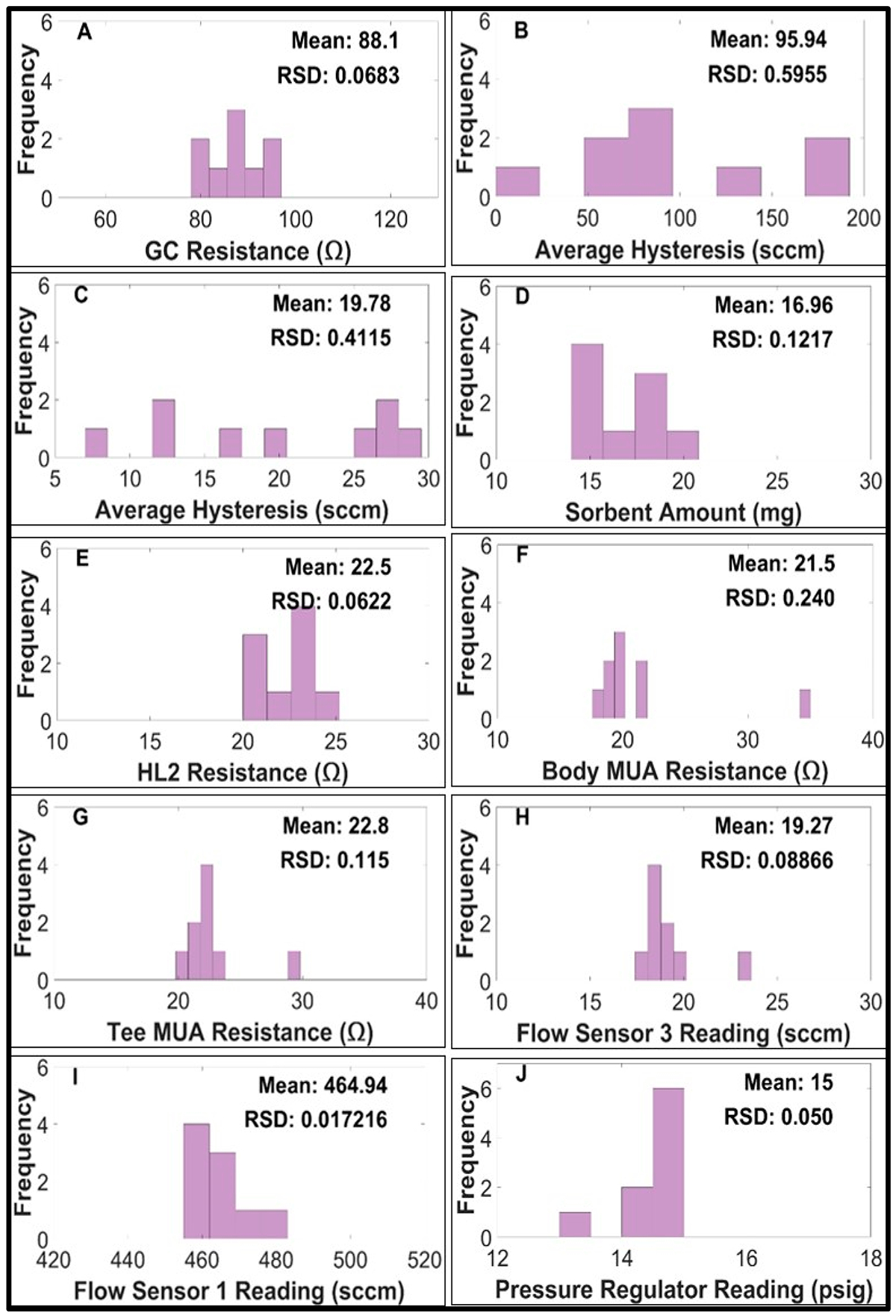
Variation of individual component test results. Each component was evaluated before being assembled into a device. Tests results include: A) electrical resistance of the gas chromatography (GC) column resistance temperature detector at 40 °C, B) the average flow hysteresis between increasing and decreasing flow curves for proportional valve 1 controlling main gas flow, C) the average flow hysteresis between increasing and decreasing flow curves for proportional valve 2 controlling Gas Chromatography Column (GC) column flow, D) the sorbent mass contained in each trap, E) the electrical resistance of the heater wrapped around the transfer line or heated line 2 (HL2) that connects the trap to the GC column, F) the electrical resistance of the heater wrapped around the flow line of the makeup adapter (MUA) that connects the outlet of the GC to the DMS detector makeup flow, G) the electrical resistance of the heater wrapped around the transfer line that delivers makeup flow to the makeup adapter, H) the flow sensor 3 (located in the desorption line) reading variation with a 20 sccm setpoint, I) the flow sensor 1 (located in the recirculation line) reading variation with a 500 sccm setpoint, J) the pressure regulator (located at the nitrogen inlet) reading variation with a 15 psig setpoint.

To minimize performance variation among devices, individual components of the flow system were subjected to testing of both electrical and mechanical properties to meet acceptance criteria prior to device assembly. Every component was externally verified for each of the 5 units prior to installation. A completed device was connected to a nitrogen cylinder as the main carrier gas to route odorants through the system for chemical analysis. The parameters and components we measured as acceptance criteria are summarized in Figure 2, and the locations can be mapped to the system level layout presented earlier (Figure 1).

#### 2.2.1. Performance of the Gas Chromatography Columns:

The GC column (Agilent, 123–1334LTM), was a 30 m coiled fused silica tubing which has an inner diameter of 0.32 mm. The inside of the tubing is coated with a sorbent material that separates odorants [13]. Odorants elute from the column at different times depending on their chemical and physical properties as well as their interactions with the lining of the column. The nitrogen flow through the GC column is controlled between 1.5–3.0 sccm. The chromatography analysis method developed specifies that the column remains at 40 °C for 1000 s in which time IPM, NPM, TBM, DMS-odorant, and ETM elute from the column and into the differential mobility spectrometer for detection. After 1000 seconds, the column is heated to 160 °C so that THT can elute and be detected.

Each GC column includes a resistance temperature detector (RTD) installed by the manufacturer (Agilent) to provide heater control feedback. To reach maximum temperature control accuracy, the RTD is calibrated by placing the column in an oven, ramping the temperature from 30 to 110 °C, and measuring the corresponding RTD resistance at set intervals. A linear regression analysis is then performed to generate a calibration slope and intercept for the given GC column. All nine GC columns were calibrated, and then their resistances were calibrated at a temperature of 40 °C, (Figure 2-A) since this is the temperature used to exhaust the first 5 odorants during the chemical detection process. The GC columns used in these devices did not introduce variation in retention times for small changes in RTD controls did not affect retention times.

#### 2.2.2. Proportional Valves:

The performance of the proportional valves (Figure 1, number12) was quantified by varying commanded duty cycle (between 0 and 4095 bits fully shut to fully open) using software in the microcontroller and then measuring the resulting system flow using both an external mass flow controller (APEX, AX-MC-500SCCM-D/GAS-N2,5M,5IN) and internal flow sensors. When testing proportional control valves, it was found that they have a significant amount of hysteresis resulting in the feedback control system needing to make large, commanded changes to control flow when varying from increasing to decreasing flow rates. In brief, the system was performing inconsistently due to a combination of sensors and high hysteresis coming from the proportional valves. The proportional valves were introducing a lot of variation in the devices and eventually were not used any more.

#### 2.2.3. Sorbent Trap:

The sorbent traps (figure 1, number3) are custom made as described earlier in the paper, [13] and are comprised of silica gel sorbent packed inside metal tubing coated in SilicoNert to avoid degradation of odorants during analysis. The trap is held at a constant 35 °C while adsorbing the sample and then is rapidly increased in temperature to 180 °C, expelling trapped chemicals, while the nitrogen carrier gas sweeps them into the gas chromatography column. The trap temperature is then to 35 °C for the remaining chemical detection process. Due to the hand-built manufacturing process, individual traps were found to contain between 15.00–20.00 mg of sorbent. The decision to use 15.00–20.00 mg of sorbent in the traps was made following industry standards for mobile detection platforms and field applications. This amount of sorbent ensures consistent operation and reliable performance. Additionally, the construction process focuses on maintaining uniformity across all traps to enhance reproducibility. Similar to the precision found in Chromatotec’s odorization monitoring devices for natural gases, biogas, and Liquefied Petroleum Gas (LPG), this approach emphasizes the importance of accuracy and reliability in chemical detection. [17]

#### 2.2.4. Heated Transfer Lines:

The device contains several transfer lines that use gas flow to direct odorants between different stages of analysis. The lines must be heated during analysis to avoid locations in the system where low temperatures can result in accumulation of chemical to the walls of the tube, resulting in decrease in signal during the analysis and contamination of signal in later runs. This is achieved by wrapping heating wire over the outer diameter of a stainless-steel tube which is insulated with shrink tubing and installing a thermocouple near the outer surface of the tubing as feedback to a thermal controller to regulate temperature. Each heated wire is individually measured to have the resistance (25Ω +/− 5Ω) needed to reach desired temperatures. All heated lines remain at a constant 100 °C during the entire chemical flow and detection process. Each heated line is tested using a temperature controller to ensure they reach desired temperatures in a 20 second window during device start-up. Variation of the heater resistances are shown in Figure 2-E,F,G.

#### 2.2.5. Flow Sensors:

The flow sensors (Figure 2-H,I) were tested using a high accuracy external mass flow controller (APEX, AX-MC-500SCCM-D/GAS-N2,5M,5IN). The purpose of this test is to verify that the flow sensors accurately measure flow rates, which is crucial to overall device performance. A specified flow rate was commanded, and the device’s internal flow sensor reading was compared to the reading given by the external, high-accuracy flow meter. The flow sensors had varying levels of accuracy which resulted in inaccurate and varying flows throughout the system. The level of error for sensor flow readings vs. actual flow rate can impact the performance and repeatability of the device, as the flow sensor provides the feedback for the flow control system, which uses the proportional valves as the flow control actuators. These sensors introduce variability not only among devices but also among measurements from the same device. Each sensor yielded different readings for the same flow rate. The inaccuracy of flow sensor 1 (figure 1, number11) which reads the makeup flow had a minor effect on variation of the detector signal amplitude because it was only 20–30 sccm. However, the inaccuracy of the desorption line flow sensor had a significant impact on the desorption flow since it was from 1–5 sccm, causing retention times to shift if the flows are not consistent.

#### 2.2.6. Pressure Regulator:

The mercaptan analyzer uses nitrogen as a carrier gas to drive odorants through the varying stages of chemical analysis. Nitrogen enters the device through a pressure regulator (Figure 2-J) to ensure the device is not over- or under-pressurized. The pressure regulator is tested by fluctuating the pressure output from the nitrogen cylinder and ensuring that the pressure in the device’s regulator remains a constant 24 psig even when the pressure at the cylinder’s regulator is greater. The set value of the pressure regulator was then increased to 38 psig to achieve the flow rates necessary for chemical detection of odorants. In Figure 2-J it can be observed that there is little variation in pressure at a given 15 psig going into the system when comparing 9 of the devices. The pressure varied from 13–15 psig with 6 out 9 devices reading a perfect 15 psig. It is not believed that this component of the device introduced variation.

#### 2.2.7. Ionization Source:

Ionization is a critical step to measure chemical analytes by the DMS detector chip. Briefly, ionization sources provide an electrical charge to mercaptan species prior to entering the DMS. As the odorant passes through the DMS detector, the charged particle has an electrical interaction with the detector pad, which results in a measurable signal that the detector records.

UV bulbs are commercially available and provide adequate ionization for the odorants in this project. We sourced a high-output 10.6 eV UV bulb (Analytical West, 510108–1061) UV bulbs have a maximum lifetime and require periodic replacement. All instruments used in this study had UV sources that were below the threshold for manufacturer published lifetime usage hours.. Their lifetime varies by manufacturer, but typically last between 10,000–40,000 hours of use. The mercaptan analyzer software logs how long the device UV bulb is on to allow for replacement as necessary. As of now, this part of the platform has not introduced variation, but as per the manufacturer, over longer use durations, it could affect signal intensity as it loses its ability to ionize the chemicals due to the decreasing intensity of the UV photons.

#### 2.2.8. Differential Mobility Spectrometer:

The Differential Mobility Spectrometer (DMS) can provide additional separation of chemical species based on the mobility of the molecules in varying electrical fields generated with an AC separation voltage, biased by a DC compensation voltage. However, the AC component of separation was set to 0V, as the GC column provided adequate separation and no AC voltage yielded maximum detector signal. Finally, the DMS chip includes detector pad and electronics to attract the charged ions and convert the resulting flow of electrical current into a sensed voltage, which can then be translated into mercaptan concentration by the AIMS software. The detailed working principles of DMS can be found in several pioneering works by Buryakov et. al. and Miller et. al. [5, 18].

The differential mobility spectrometer (DMS) is assembled into its fixture and tested prior to installation. For verification, a sample of THT mercaptan at 1000 ppm is prepared in a Tedlar bag. The DMS is then connected to a LabView data acquisition system that controls electrode voltages and reads the detector electronics. Then, a heated metal tee is connected to the DMS’s inlet to mix nitrogen and THT, replicating the makeup adapter in the chemical detection platform. THT at a concentration of 1000 ppm is injected using a syringe pump (KD Scientific, 789100A) along the flow path at a rate of 12 mL/min to then be mixed with nitrogen coming into the tee at a 90-degree angle at 500 sccm. Mixing the nitrogen and the THT at these rates will result in a final concentration of 1 ppm when it reaches the detector pad in the DMS. This setup is presented in supplemental figure 2.

When there is no sample going through the DMS other than nitrogen carrier gas, the detector electronics provide a signal baseline of 2.50 V. To determine if the DMS is functioning properly with adequate sensitivity, we expect a voltage drop from this baseline of 0.15–0.20 V when THT mercaptan is introduced and the ionized THT molecules begin impacting the detector pad. Once it is determined that the DMS had adequate sensitivity, data is recorded for 3 minutes while the THT-nitrogen mixture is going into the DMS continuously. This process is repeated three times to ensure repeatability.

Each DMS device consists of interface board, main board stack, chip with flow path and electrodes, and ionization UV bulb. The detector board was the source of variation on whether the DMS device would produce a signal, and, if so, what the signal/concentration gain would be. The 50 GΩ gain resistor (Mouser, 279-RH73X2A50GNTN) which was part of the Op-amp based current/voltage conversion on the detector board for the DMS had a +/− 30% error which meant it could have a resistance range of 35–65 Ω. If the resistance were to be in the lower end of the range, the signal will be much lower, while if the resistance were to fall in the higher end, it would result in higher signal.

**Figure 3:**
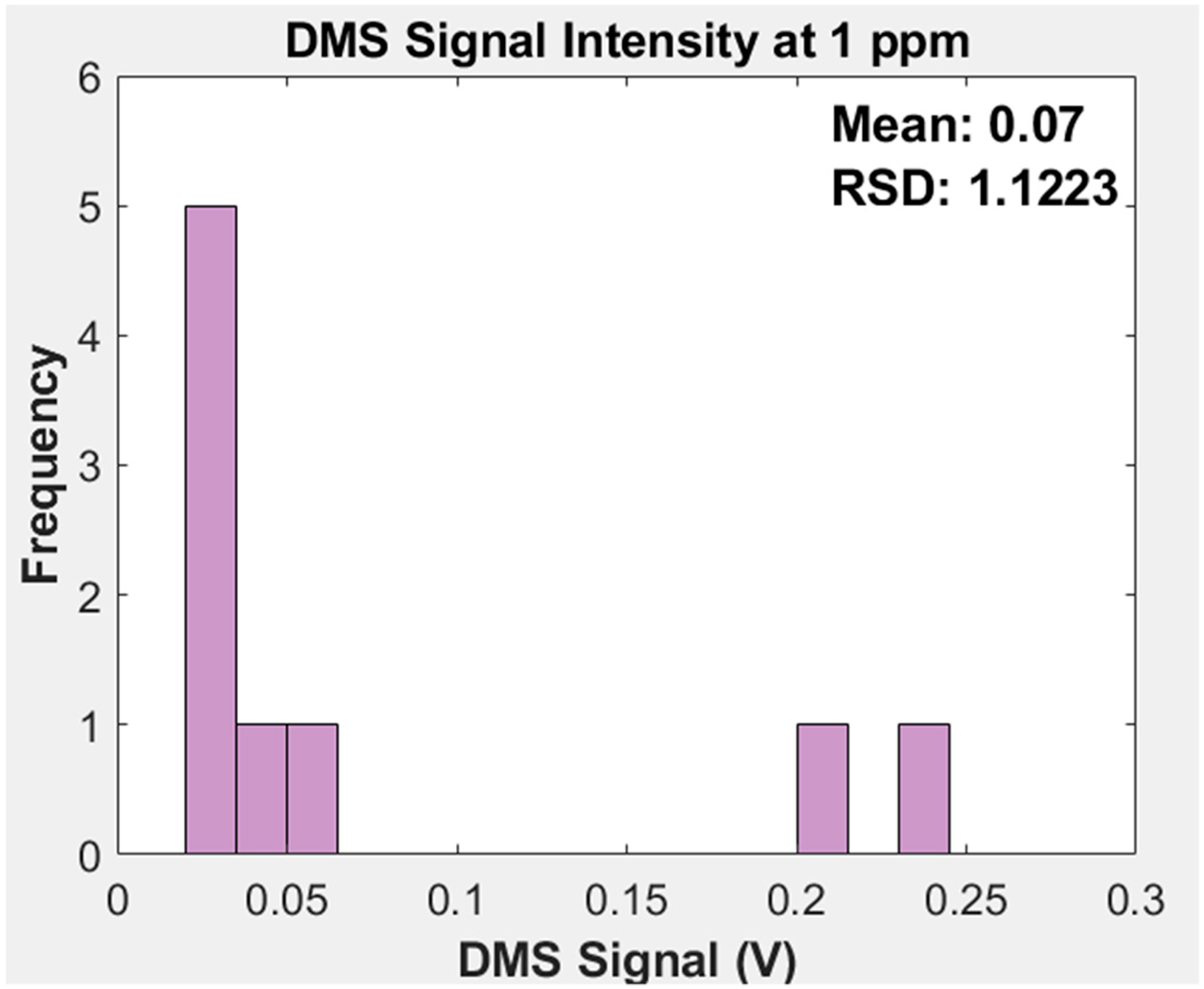
Differential Mobility Spectrometer (DMS) signal intensity variation of THT at 1 ppm in a balance of N_2_ for the nine DMS units that were evaluated.

Figure 3 depicts the range of signal intensity observed when testing DMS units. The data points in the figure represent different DMS units with unique hardware components: a different UV ionization source, chip, fixture, and electronics boards. No DMS device that we produced was identical when converting analyte concentration to signal intensity. Despite the individual parts are purchased from the same commercial manufacturers and circuit boards were assembled by professional suppliers, the overall DMS complexity resulted in unanticipated variation in DMS performance. Many combinations of components produced no measurable signal. We determined that the signal output must be at least 0.02 volts at the 1 ppm concentration for the DMS to pass this quality check. A DMS with less than 0.02 volts when installed into the device would not detect any of the odorants.

## 3. Results

### 3.1. Device Failure Modes:

While in section 2.2 we described component-level testing prior to device assembly, this section summarizes our efforts to produce five fully assembled devices, and our approach to troubleshooting the issues that were observed during assembly.

When testing the devices, one or more presented failure symptoms that caused delays in the production process. Each of these issues were recorded along with our work to address the root problem.

#### 3.1.1 Failure mode A: Gain resistor

As previously mentioned in the discussion of the DMS, the gain of the ion detector amplification circuit is critical to achieving proper signal for a given concentration of analyte. Our baseline design of this circuit specified a 50 GΩ resistor as the feedback resistor for the primary Op-amp (Supplemental Figure 2). Unfortunately, these surface mount components have no identifying marks, and, during our assembly, process errors resulted in mixing stock of the 50 GΩ resistors with 10 GΩ versions. Once soldered into place, there is not a convenient, accurate method to measure the resistance of such a high resistance component (cannot be measured with a standard lab multimeter). As a result, we produced some versions of our DMS with the wrong gain, but some of these systems met the 0.02 V at 1 ppm DMS signal quality check and were built into functional units. Only after comparison to performance of other units, disassembly, and development of a method to measure such high resistances were we able to determine this failure mode due to our assembly quality issues.

#### 3.1.2 Failure mode B: Sorbent trap

The traps are made to have anywhere between 15.00–20.00 mg of sorbent which is measured utilizing a high accuracy scale. Most traps contained roughly 20 mg of sorbent. Though testing and developing on the traps had been done prior to using them in the chemical detection platform, it was observed that lower sorbent amounts impact signal intensity. Figure 3-B shows the signal decrease due to using a trap that contained 15 mg of sorbent.

#### 3.1.3 Failure Mode C: Feedback controlled system failure resulting from the inherent inaccuracies of flow sensors and hysteresis of proportional control valve

The chemical detection platform was originally controlled using a feedback-controlled system which relied on the readings from the flow sensors (Honeywell, HAFBLF-0750C4AX5, HAFBLF0050CAAX5) to operate the proportional valves (Norgren, D170.0004). These resulted in inconsistencies in the flow, specifically in the desorption line which controls the retention times of each on the odorants. Figure 3-C shows the resulting chromatographs with inconsistent results from varying flow rates. Variation in desorption flows impact the retention time of chemicals in the GC column and the sensor’s ability to identify and distinguish individual chemical species. To achieve consistent retention times across multiple devices, desorption flow rates varied from 1.2–3.0 sccm. Thus, a small variation in flow can result in significant changes in retention times. A variation of only 0.5 sccm can change elution times by about 200 seconds. This can be shown in supplemental figure 3.

Further investigation into the flow sensor accuracy issue revealed that the specifications for this device require that their maximum operating pressure was 25 psig, above which they do not meet their published accuracy specification. Development of the initial prototype was performed within this pressure limitation. However, it was identified that a more aggressive GC column is required to separate all mercaptan chemical species that appear in natural gas, some of which are created during gas processing, during field testing. Consequently, the selection of a more effective GC column required operating pressures of 38 psig to achieve target desorption and recirculation flow rates, meaning the flow sensors were operating in conditions outside of their manufacturer specification.

#### 3.1.4 Failure Mode D: Leaks

Any leak in the flow system would either decrease the device sensitivity, impact its reproducibility, or completely prohibit analysis. Each device is extensively leak tested to ensure flow systems are completely sealed. To complete a leak test, helium is connected directly to the carrier (i.e., nitrogen) inlet of the device. After waiting 3 minutes for helium to circulate through the device a commercial helium leak detector (RESTEK, 28500) it can be verified that there is flow through the whole system by obtaining a reading at the outlet of the DMS. Finally, to verify that there is no leak, each connection, corner, and line of the flow system is tested using the leak detector. As explained before, the desorption section of the flow system is operated at very low flows, 1.5–3.0 sccm. This section of the flow system is the main driving force that will impact retention times if it were to vary. A small 0.5 sccm leak can have a similar impact on retention times to a change in desorption flow setting.

#### 3.1.5 Failure Mode E: DMS Chip Failure

DMS chips were manufactured at the CNM2 class 100 cleanroom facility at UC Davis. For ionization of analytes, a window must be drilled into the borosilicate substrate for UV light to pass through. Larger windows result in more ionization power, and more sensitivity; but because detector chips are made of glass, larger were more prone to cracking, resulting in leaks and substantial signal loss. Occasionally, chips would contain microscopic cracks but pass visual inspection and leak checking, but cracks propagated over time, which slowly impacted detector signal over time (Figure 3-E). Drilling smaller ionization windows prevented cracks from forming, but the size did not provide adequate ionization and resulted in poor device performance.

### 3.2 Management and Outcome

**Figure 4:**
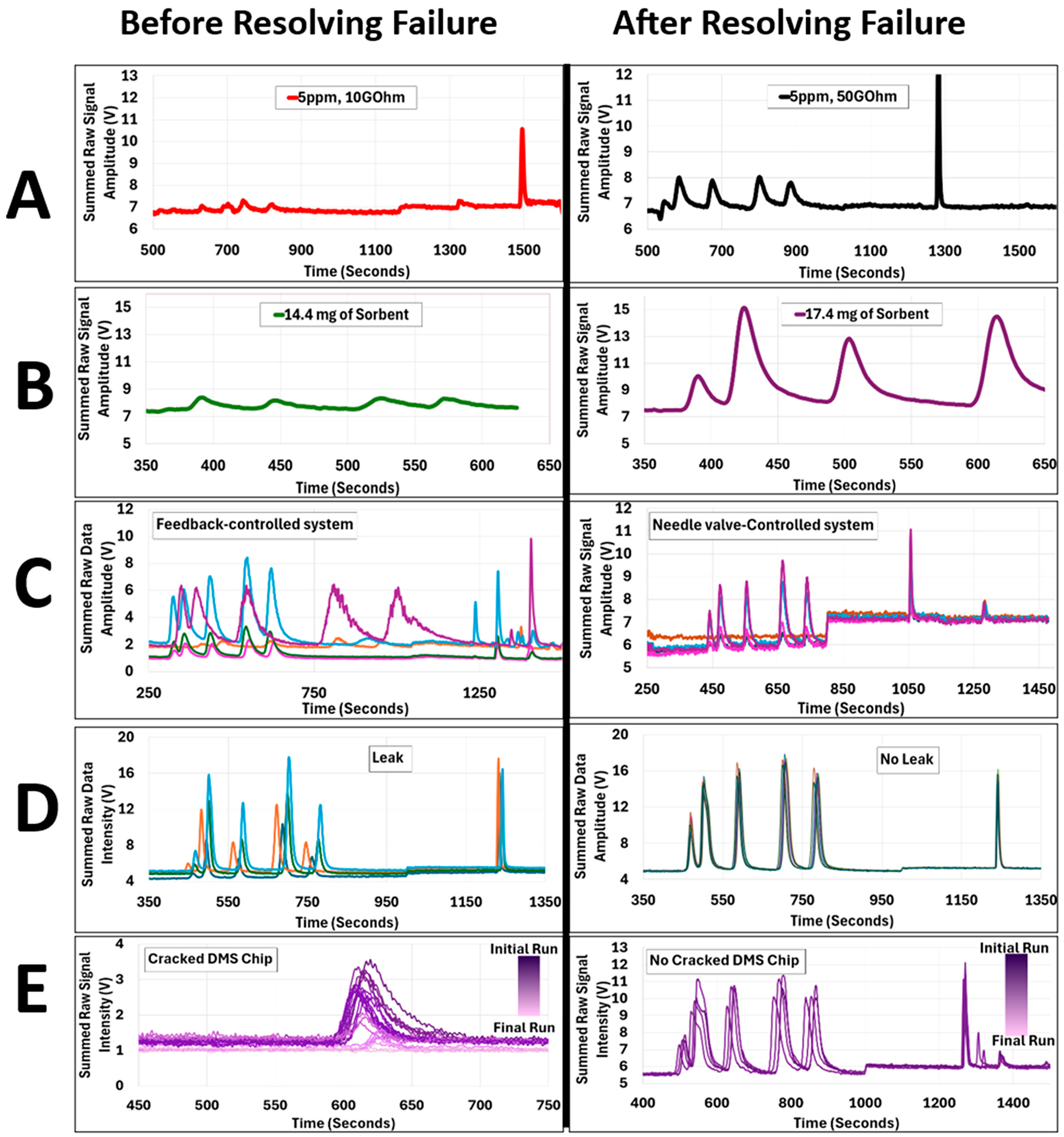
Chromatographs showing before and after resolving the failure modes present in one or more devices. A-Before: (N=1) is the signal intensity resulting from using a 10 GΩ gain resistor. A-After: (N=1) is the resulting increase in signal due to using a 50 GΩ gain resistor. B-Before (N=1) is the chromatogram showing low signal due to using a trap with 15 mg of sorbent. B-After (N=1) is the resulting increased signal from using a trap containing 17 mg of sorbent. C-Before (N=5) is the resulting variation in retention times from having an electronically controlled system. C-After (N=5) is the result from swapping over to a needle-valve controlled system. D-Before (N=4) is the resulting variation in retention time due to a leak in the system. D-After (N=5) after is the result from identifying the leak and fixing it. In this case the device gave extremely consistent data that all 5 runs at 5 ppm are stacked on top of each other. E-Before (N=21) is the resulting decrease in signal over time resulting from a cracked chip. E-After (N=4) is the resulting consistent signal intensity from a chip with no cracks. Graphs are plotted with different axis just for clarity.

In Figure 4-A we compare the signal intensity of the DMS detector with two different gain resistors (10 GW and 50 GW) and we observed a 15% increment of signal intensity for samples with a concentration of 5 ppm.

Figure 4-B depicts the resulting increase in signal at a 5 ppm concentration of the first 5 mercaptans after swapping the sorbent trap containing 15 mg to one containing 17 mg. The 4-B-Before chromatograph showed low sensitivity with a sorbent trap containing 15 mg of sorbent. While utilizing a trap with 17 mg of sorbent dramatically increased the sensitivity as shown in figure 4-B-After of the device as more sample was able to be pre-concentrated into the sorbent. In this case the trap was the focus because the devices that had been previously built all had sorbent traps that contained 16.00+ mg of sorbent and yielded discernible signals for varying sample concentrations.

Figure 4-C-Before demonstrated the inconsistent behavior of the flow system while using a feedback-controlled system. As explained before, the flow sensors have a significant amount of error, and the proportional valves have substantial amount of hysteresis. These issues combined caused inconsistencies with retention times (the time it takes for each mercaptan species to exhaust from the GC column and reach the detector). The prediction algorithm depends on a consistently performing device, as it expects each mercaptan species to be detected within a 35-second window. As a minor alteration in flow rate, as small as 0.5 sccm, led to a significant 200 second shift in the retention time. As a remedy, the feedback control with proportional valve is replaced with a needle valve-controlled flow system, which is tuned manually.

Figure 4-C-After demonstrates the repeatability of the device after the flow system was modified to use manually controlled needle valves. The selected were chosen to operate optimally at low flow rates, e.g., 1.0 – 300 sccm. Initially the device is set to 1.3 sccm desorption flow and 550 sccm recirculation flow, which are the minimum flow rates for device operation. Then, we conducted an initial run of the device with a standard mixture of mercaptans to know relative RTs of target chemicals. We target specific retention times for each mercaptan species to ensure consistency across all working devices. Depending on the elution times seen in the generated from the initial run, needle valves can be tuned to accelerate or slow down mercaptan elution to meet specification. If the mercaptans are eluting later than desired, flow rates are increased, and if peaks are eluting earlier and/or have inadequate separation, flow rates are reduced. Special attention was given to mercaptans ETM and DMS-odorant, the first two eluting compounds, as faster flow rates prevented these peaks from separating. Flow is tuned until there is a 5–10 second window between them so that the prediction algorithm can determine concentrations more accurately. Through the optimization of signal output, we have observed variations in elution times and flow settings across different devices. This indicates that uniform optimization does not result in consistent performance metrics among all devices. This emphasizes the importance of adequate flow tunning parameter for each device.

Figure 4-D shows the resulting chromatographs from before and after having a leak in the system. Leaks impact the resulting data in two ways: (1) signal reduction and (2) varying retention times. The symptoms for a leak are also present in all of the other failure modes. Therefore there’s no unique symptom indicating a leak. A leak in the recirculation line will only result in decreased sensitivity whereas a leak in the desorption line will result in inconsistent retention times and decreased sensitivity.

The last failure mode before and after being fixed can be observed in Figure 4-E. When a chip begins to crack, the resulting signal diminishes. If cracks propagate over time, the signal will continue to drop. In this case a 5-ppm sample using NPM was used to carry out this study. This was done to observe the behavior of a device over a period of 3 days while only using one chemical to shorten the chemical analysis time. Figure 4-E-Before, shows a deteriorating signal over time. When there are no cracks in the DMS Chip the signal intensity remains consistent as shown in figure 4-E-Before.

## 4. Discussion

Chromatographic technology, while highly effective for gas detection, presents several key technical and scientific challenges that must be addressed to ensure reliable performance. One of the primary issues is achieving high-resolution separation, which is crucial for distinguishing between similar chemical species, such as different mercaptans. Additionally, maintaining sensitivity to detect low concentrations of VOCs is essential, particularly in applications requiring precise monitoring and control. Stability and accuracy over extended periods are also critical concerns, as fluctuations in these parameters can lead to inconsistent results and reduced reliability. Our approach leverages the industry-standard chromatography, coupled with our solid-state detector, to address these challenges effectively. This combination not only enhances specificity and sensitivity but also meets the stringent requirements of utility companies for individual measurements of each mercaptan species. By focusing on these key issues, we aim to provide a robust and reliable solution for portable VOC detection.

**Figure 5:**
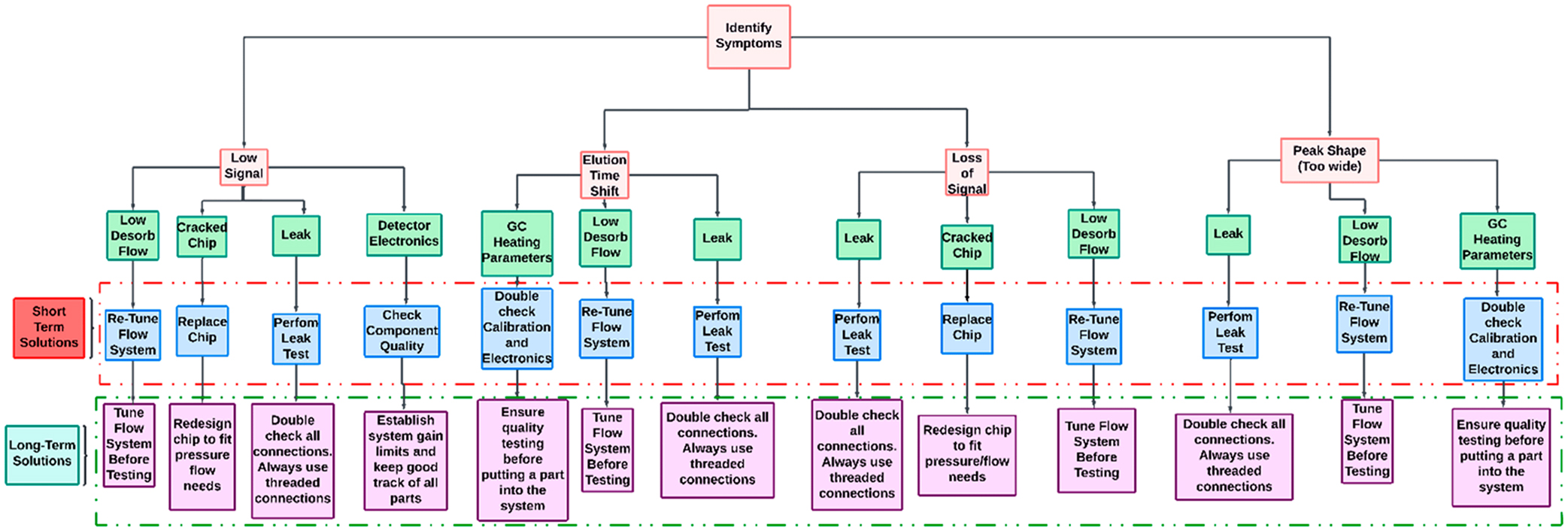
Flow chart diagram describing which course of action to take to diagnose a chemical detection device.

Through this work, we hope to provide troubleshooting guidelines to other teams assembling multiple highly integrated VOC detection platforms using sorbent traps, gas chromatography columns, and chemical detector. Trying to replicate a device many times over has proven to be quite challenging. Figure 5 depicts a flow chart summarizing each of the failure modes described in this section and corresponding plausible remedies. The goal is to provide a quick visualization of each of the failure modes as well as a short- and long-term solutions for each of them.

The first step when trying to troubleshoot a device is to identify the “symptoms” (issues) present. In the case of this project there were 4 main issues: (1) low signal, (2) retention time shifts, (3) loss of signal, and (4) peak shape/width. Symptoms were resolved with a short and long-term solution.

### 4.1 Symptom: Low signal

If the issue of low signal were to arise there are four possible causes we have identified: (1) low or no desorption flow, (2) cracked DMS chip, (3) a leak in the flow path, and (4) detector electronics issues. To resolve low desorption flow, a short-term solution is to re-tune the flow system, but to avoid this it is important to follow the flow tuning process before any chemical detection can be done. This process can be lengthy because each device required a different desorption/recirculation flow combination to output the desired signal. Low signal can also be present if there is a cracked DMS chip. As shown before in Figure 4-E, when a chip crack starts to propagate, the signal will decrease over time. A quick solution for this is to replace the DMS chip. In our case, to avoid cracking in future DMS detector chips, there should be redesigned without needing to drill the ionization window into the borosilicate glass substrate.

A leak in the flow path will reduce signal by the loss of sample as it is making its way to the detector. A leak test as described earlier is the easiest way to identify a leak and solve it quickly. However, a more permanent solution is to perform leak test routinely before doing any chemical testing to avoid delays.

Finally, detector electronics issues can alter the intended gain of the system (ionized analyte flow rate vs. signal voltage). Establishing an overall system gain quality check with a pass/fail limit enables identification of low gain systems and can trigger more thorough inspection and component quality checks to identify the source of the issue. However, establishing a quality check process with a series of component tests for each electronics board and critical electronic components to prior to assembly would reduce the re-work required by failures in system level assessments. For even with QA/QC parameters in place it can’t be guaranteed that the device will perform adequately.

### 4.2 Symptom: Elution time shifts

The second symptom present when troubleshooting these chemical detection platforms was elution times shifts. We identified three root causes for retention time shifts: (1) inconsistent heating control of the gas chromatography column, (2) low desorption flow, and (3) leaks. To resolve GC heating parameters, it is essential to investigate the calibration of the GC column heating system and the RTD feedback for temperature control.

Low desorption flow can also lead to inconsistent retention times due to the inability for the system to fully pressurize in a reasonable amount of time. To avoid this issue the flow system should undergo flow tuning before carrying out experiments.

A leak can also shift retention times. This is due to the sporadic behavior a leak can have, meaning exterior factors can cause the leak to be greater or smaller at different ambient condition. A leak test can help to quickly solve this issue, but a more permanent solution is to routinely perform leak testing before performing any chemical analysis and using proper threaded connections.

### 4.3 Symptom: Loss of Signal

We observed loss of signal can happen due to three main causes: (1) a leak in the flow system, (2) cracked chip, and (3) low desorption flow. Leaks tend to be a recurring issue when assembling devices. In this case if there is a leak in the desorb line and most of the flow is coming out from there, then all the chemical sample will be lost to the ambient and unable to reach the detector.

A cracked DMS chip was also observed to result in loss of signal. Based on our experience, not only repetitive use of the DMS chip can lead to crack initiation and propagation, but also changes in ambient conditions can facilitate those processes. The presence of cracks may not present a deterioration in signal immediately. Thermal or physical stress enlarge existing cracks and rapidly signal quality. Sometimes cracks propagate rather quickly for a change in ambient condition (mostly overnight), and signal gets lost rapidly as well. Cracks can also slowly propagate, reducing detector signal slowly over time. To resolve the issue quickly the DMS chip can be replaced, but a more permanent solution can be implemented by re-designing the chip to prevent cracks from occurring by eliminating drilled holes and therefore reducing stress points.

### 4.4 Symptom: Peak Shape / Width Variation

Peak shape is an important aspect to analyze mercaptan concentrations. Ideally, chemical peaks in GC data should be sharp, symmetrical, and Gaussian shaped.[19–21] As peak width increases, quantification of the chemical concentration become increasingly difficult due to a possible overlap of different chemical signals. In our instance, several mercaptan compounds had relatively close retention times from the GC column. If these peaks were unacceptably wide, two mercaptan species could merge (i.e., not resolve) in the resulting data, conflating concentration readings. This was particularly a problem between mercaptans ETM and DMS-odorant because they elute from column close to one another at around 370 seconds and 410 seconds respectively.

A leak can be one of the causes for peaks broadening. This would be specifically a leak in the desorption line. This issue can be fixed using a leak test. Too low of a desorption flow can also broaden peaks because the carrier gas is too slow when carrying analytes through the system. This issue can be resolved by tuning the system to optimal flows, and to avoid this recurrence, flow tune the system before doing any chemical testing in the device.

Finally, peaks can be too wide if the GC temperature is not consistent or too high or too low. If the column is too hot, analytes may co-elute, resulting in shoulder peaks or peak splitting. Conversely, when the column is too cold, analytes linger in the GC column for a longer period of time and disperse, broadening the resulting peak as they reach the detector. Implementing quality checks on all testing will avoid issues with inconsistent performance of individual parts.

## 5. Conclusions

Developing a chemical detection platform is not an easy task. It takes a great deal of planning and part interfacing to have a working prototype. Building multiple chemical detection systems presents an entirely different set of challenges due to the variability each individual component sub-system brings into the project. Although most components likely will not have significant variability, some are more sensitive and less perceptible component variability can significantly impact the behavior and data quality between device system outputs. By sharing our approach to troubleshooting and failure resolution, we hope others now have more guidance in building multiple chemical detection platforms.

In our experience, meticulous attention to detail during the assembly and calibration phases is crucial for ensuring consistency and reliability across all units. Continuous monitoring and iterative testing are essential to identify and mitigate any discrepancies that arise from component variability. Additionally, implementing a robust QA/QC process helps in maintaining high standards and reducing the potential for errors during production.

Despite our successes, there remain several areas for future improvement. Enhancing the sensitivity and selectivity of the DMS detector will be crucial as we expand the range of detectable chemicals. Designing a flow system that provides the adaptability of the original closed loop control with the stability of the needle valve control system is required.. Moreover, automation of the QA/QC processes could streamline production and minimize human error, making large scale deployment more feasible.

By addressing these challenges and continuously improving our quality process, the ultimate goal is to develop reliable, high-performance detection systems that can be deployed across a wide range of applications, from environmental monitoring to industrial safety and others in the field, fostering innovation and collaboration in the development of the next generation chemical detection platforms.

## 6. Patents

Not applicable

## Supplementary Material

Supplemental Material

## Data Availability

The raw data supporting the conclusions on this article will be made available by authors upon request. The software code and circuit designs are available on GitHub for non-commercial use. Please refer to Professor Cristina Davis’ webpage for more information. This material is available as open source for research and personal use under a Creative Commons Attribution-Non Commercial-No Derivatives 4.0 International Public License (https://creativecommons.org/licenses/by-ncnd/4.0/). Commercial licensing may be available, and a license fee may be required. The Regents of the University of California own the copyrights to the software. Future published scientific manuscripts or reports using this software and/or hardware designs must cite this original publication (DOI: xxxxxxxxx).
