# Supplementary material for: A Diagnostic Case Study for Manufacturing Gas-Phase Chemical Sensors
Source: Chemosensors (Basel). Author manuscript; Available in PMC 2025 Sep 24. (PMC12456322; doi:10.3390/chemosensors12080155)
Supplement: Supplemental Material [file NIHMS2069439-supplement-Supplemental_Material.zip › chemosensors-3049238-supplementary.pdf]

Supplementary Materials:

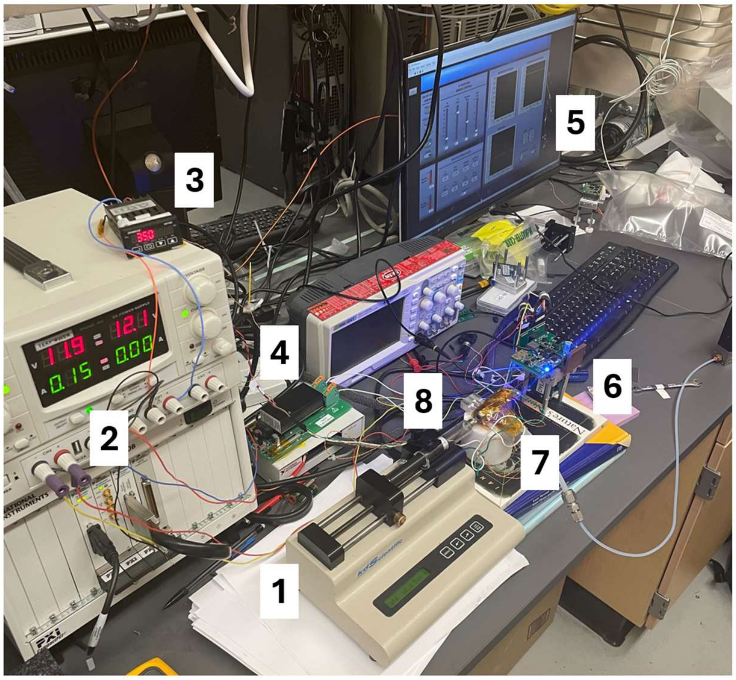

Figure S1. The DMS testing setup is composed of the following: (1) a syringe pump used to inject the chemical sample at a constant rate, (2) a bench power supply controlling the voltages of the DMS electronics and the high-voltage power supply (HVPS), (3) a temperature controller to heat the mixing tee, (4) an HVPS that powers the UV ionization bulb in the DMS, (5) the LabView program used to control all the boards in the DMS, (6) the DMS units under test, (7) the mixing tee being heated to avoid cold traps, and (8) the glass syringe containing the chemical analyte THT at a 1000 ppm concentration.

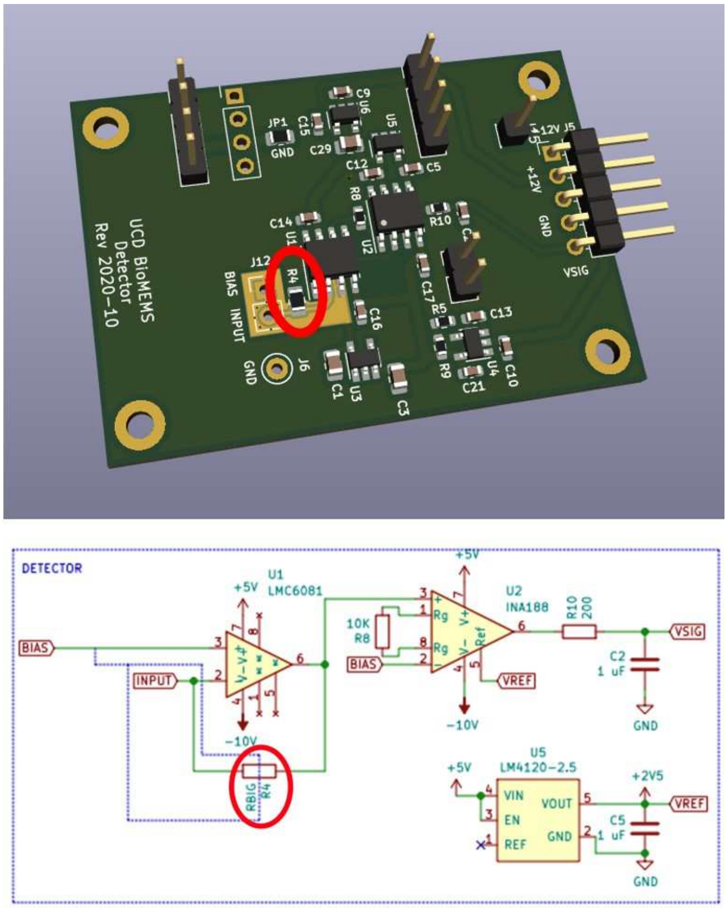

Figure S2. Detector board diagram showing where the gain resistor is located.

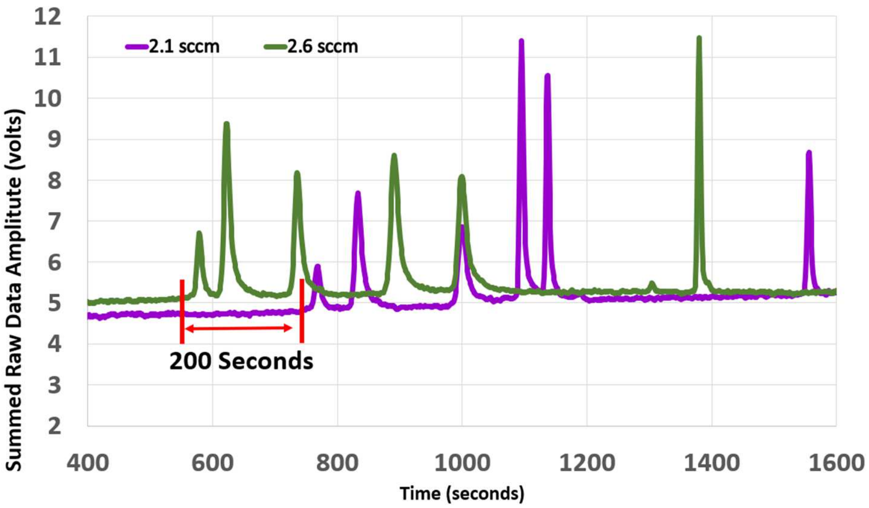

Figure S3. Chromatograph showing the impact a 0.5 sccm change in the desorption flow can have on retention time.
